# Effectiveness of Autogenic Drainage in Improving Pulmonary Function in Patients with Cystic Fibrosis

**DOI:** 10.3390/ijerph20053822

**Published:** 2023-02-21

**Authors:** Magdalena Żak, Hugues Gauchez, Marek Boberski, Anna Stangret, Agnieszka Kempinska-Podhorodecka

**Affiliations:** 1 Physiotherapist’s Office Children’s Therapy in Szczecin, 71-502 Szczecin, Poland; 2 Centre de Kinésithérapie Respiratoire et Fonctionnelle du Nord in Marcq en Baroeul, 59700 Marcq-en-Barœul, France; 3 Institute of Respiratory and Neurodevelopmental Support for Children in Szczecin, 71-502 Szczecin, Poland; 4 Department of Medical Biology, Pomeranian Medical University in Szczecin, 70-204 Szczecin, Poland

**Keywords:** autogenic drainage, cystic fibrosis, Simeox device, chest physiotherapy, airway clearance techniques, respiratory rehabilitation

## Abstract

The use of autogenic drainage (AD) in patients with cystic fibrosis (CF) has been officially approved; therefore, the purpose of this study was to compare the efficiency of the leading therapeutic techniques based on AD in patients with CF; Among patients with CF assessments were made of spirometric parameters, percent blood oxygen saturation, and the general feeling of the patients (Borg, VAS, and mMRC dyspnea scale) before and after therapy using AD, using AD in connection with a belt or a Simeox device and AD in combination with both a belt and Simeox device simultaneously. The best therapeutic effects were generated by the combination of AD with the belt and with the Simeox device. The greatest improvements were observed for FEV1, FVC, PEF, FET, saturation, and patient comfort. In patients <10.5 years of age, the increase in the level of FEV3 and FEV6 was significant in comparison to older patients. Due to their efficacy, therapies connected with AD should be applied not only in hospital departments but also during daily patient care. Given the particular benefits observed in patients <10.5 years of age, it is important to guarantee real accessibility to this form of physiotherapy, especially in this age group.

## 1. Introduction

Cystic fibrosis (CF) is a genetic disease that interferes with proper lung ventilation and is associated with a chronic stasis of secretion [1]. It is a complex multi-organ disease, with an 85% mortality rate that can be directly related to respiratory system complications.

Continuous physiotherapy, including autogenic drainage (AD), is essential in the treatment of patients with cystic fibrosis.

AD therapy was first administered to patients with asthma and only later expanded to other disease associated with the overproduction of secretions in the bronchial tree—including cystic fibrosis (CF) [2]. Chevaillier [2] defined AD as a therapeutic approach based on the optimization of the physiological relationships in the respiratory system to remove lingering secretions caused by pathophysiological mechanisms. The method was approved at the Conférence de Consensus de Lyon in 1994 [3,4] and is supported by the International Physiotherapy Group for Cystic Fibrosis [5].

While AD is a technique used in general practice, there are a very limited number of scientific works evaluating its effectiveness. AD is comparable to other respiratory cleansing techniques and can be considered as an alternative technique in the target patient group. Therefore, it is important for this technique to be reviewed to prove its effectiveness in patients with cystic fibrosis [6].

AD is an innovative technique for clearing the bronchial tree that is based on controlled breathing, by which expiratory airflow mobilizes secretions from the small bronchi to the central bronchi. The secretions are displaced and removed by adjusting the intensity and speed of breathing. Secretion evacuation is based on two different mechanisms: mucociliary clearance and the effect of airflow forces [2,7]. According to the biomechanics of the chest and diaphragm, inspiration should take place in three dimensions. At its peak, air is held for 2–4 s with the glottis open. This inspiratory pause affects the homogenization and uniformity of air distribution and prevents bronchial compression [8].

Given the complexity of the problems associated with CF, and the dynamics of the disease, the primary aim of this study was to compare the effectiveness of leading therapeutic techniques—based on AD—that are used separately or in combination in patients with CF. Most previous studies have analyzed the effectiveness of physiotherapeutic techniques after completion of the therapeutic cycle [9,10,11,12,13,14,15]. Until now there have only been a few studies that evaluated the effect of autogenic drainage in which spirometric parameters were examined immediately after therapy [16,17,18]. In this study, we collected data before and immediately after therapy which is crucial in assessing the effectiveness of any related technique. The effectiveness of individual respiratory therapies was analyzed based on spirometry and blood oxygen saturation testing, as well as fatigue and dyspnea intensity rating scales.

## 2. Materials and Methods

### 2.1. Patient Characteristics

Among 12 CF patients, aged 14 ± 12 years, a total of 48 controlled trials were completed over a period of 12 months (each of the 12 studied patients has received 4 different therapies). Spirometric parameters and dyspnea and fatigue scales were assessed before and after each of the following four therapies applied individually: (i) AD; (ii) AD in connection with a belt (AD + BELT); (iii) AD in connection with a Simeox device (AD + SIMEOX); (iv) AD in connection with both a belt and the Simeox device (AD + SIMEOX + BELT). 

Inclusion criteria for the study were individuals diagnosed with CF that had respiratory stability and no active oxygen therapy. Exclusion criteria were: being under the age of 5 years (due to inability to cooperate with spirometry testing); percent blood oxygen saturation at rest below 94%; a history of pneumothorax or hemoptysis in the last 6 months; exacerbation of broncho-pulmonary disease; and intravenous antibiotic therapy. Each participant provided their written informed consent.

Approval for the study was obtained from the Bioethics Committee of Regional Medical Chamber in Szczecin, No. 07/KB/VII/2019, dated 13 June 2019.

### 2.2. Applied Respiratory Therapies

AD was optimized by positioning the patient to enable ventilating as much of the lung area as possible. Thus, drainage was conducted in sitting and semi-recumbent positions and while the patient was lying in the side position. During the AD procedure, coughing was controlled by the patient, and the upper respiratory tract remained constantly open. The patient’s head was positioned in a slightly inclined position, so as not to limit respiratory flow. To standardize air distribution, a slow inhalation was made in all areas of the lungs, followed by a sustained inhalation lasting 2–4 s, and then a passive exhalation with a descending, relaxed chest, and perceptibly warm exhaled air with no resistance. The air was exhaled through a mouthpiece to minimize air resistance. Using manual resistance from a therapist, air flow in the bronchial tree was modulated to target lower tidal volumes. Therapy time was adjusted individually, but it did not exceed 30 min per patient.

A belt placed on the chest near the lower part of the rib cage was used to support AD. The belt allowed for the stimulation of the lung areas that are more difficult to ventilate during normal breathing and allowed the chest to move smoothly during asynchronous chest work. The positions, timing of the therapy, and placement of the therapist’s hands were the same as when applying autogenic drainage alone. The patient kept the mouthpiece in his mouth while performing both techniques. The patient and his family were instructed on how to properly use the belt to continue the therapy at home.

AD was also supported by a Simeox device [19]. The action of the device under the influence of a specific impulse changes secretion consistency. With the application of optimal air vibrations, the mucus, due to its thixotropic properties, liquefies in less than 2 s, but quickly returns to its initial form when the impulse stops [20,21,22]. When using the Simeox device, the patient’s inhalation takes place beyond the device’s function, while exhalation is correlated with the device by activating a button on the device (or remote control) that triggers an action that does not allow the airway to collapse and the secretions to return to their original consistency [23]. Operation of the Simeox device followed the guidelines in the instrument’s manual and content provided at a certified training.

### 2.3. Spirometric Examination and Saturation

Spirometric examinations were conducted with a Spirolab NEW device (MIR via del Maggiolino 125, 00155 Roma—Italy), and the following respiratory parameters were recorded: forced vital capacity (FVC); forced expiratory volume in one second (FEV1); forced expiratory volume in 3 s (FEV3); forced expiratory volume in 6 s (FEV6); peak expiratory flow (PEF); and forced expiratory time (FET). Spirometry was performed 20 min before and 20 min after therapy. A Sanitas pulse oximeter test was conducted using the ring finger of the right hand. 

### 2.4. Rating Scales for Dyspnea and Fatigue

Both before and after therapy, patients assessed their fatigue levels using the Borg Rating of Perceived Exertion Scale, the Visual Analogue Scale (VAS), and the modified Medical Research Council (mMRC) dyspnea scale [24,25,26]. Importantly, previous studies have confirmed the effectiveness of using these scales in children [27,28,29].

### 2.5. Statistical Analysis

Statistical analyses were conducted using STATA 11 statistical software (StataCorp, College Station, TX, USA). Quantitative variables were described by the arithmetic mean (x), standard deviation (SD), minimum (Min), maximum (Max), median (Me), quartile Q25, and quartile Q75. Qualitative variables were presented as frequencies (%). Differences between the means assessing the effect before and after the therapy were evaluated using the Student’s *t*-test when the variables had a normal distribution and the Wilcoxon signed rank test with non-normal data. In the analysis, the Kolmogorov–Smirnov test and the Mann–Whitney test were also performed. For all analyses, *p* < 0.05 was considered significant.

## 3. Results

### 3.1. Analysis of Respiratory Parameters

**Figure 1:**
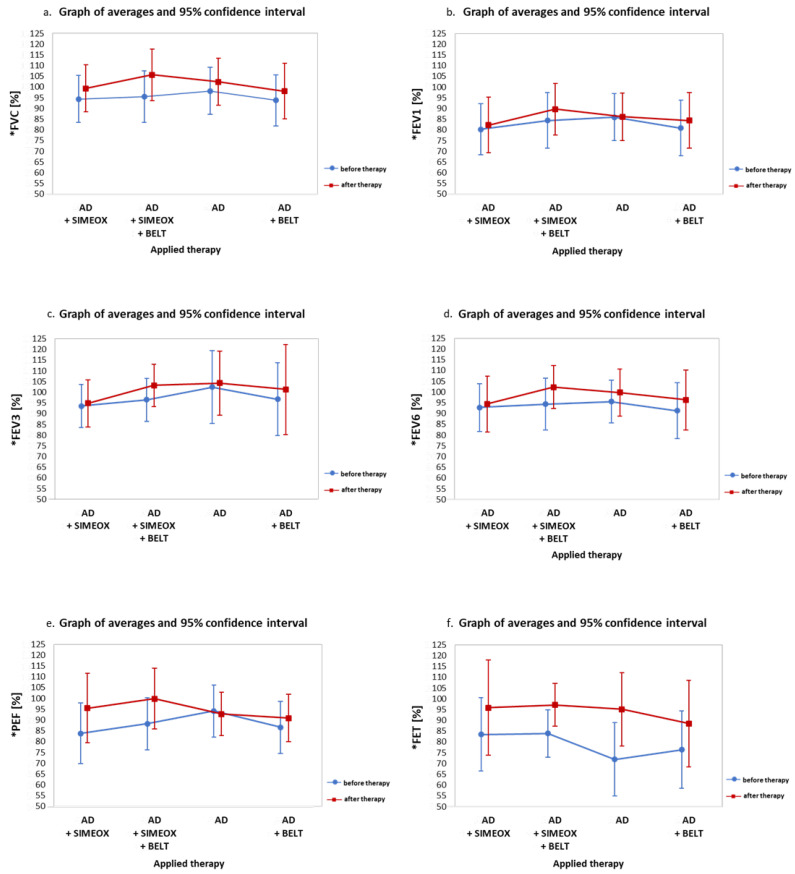
Graphic representation of (**a**) forced vital capacity (FVC); (**b**) forced expiratory volume in 1 s (FEV1); (**c**) forced expiratory volume in 3 s (FEV3); (**d**) forced expiratory volume in 6 s (FEV6); (**e**) peak expiratory flow (PEF); and (**f**) peak expiratory time (FET) before and after therapy. * AD, autogenic drainage.

Following AD, both alone and with the support of other therapies, FVC significantly increased (Figure 1a). There was an increase of 10 ± 9% after AD in combination with both the belt and Simeox device (96 ± 17% vs. 106 ± 18%; *p* = 0.005). An FVC increase of 5 ± 4% was observed after AD with the Simeox device (94 ± 16% vs. 99 ± 16% (*p* = 0.004)); a 4 ± 4% increase in FVC after AD alone (98 ± 17% vs. 102 ± 17% ((*p* = 0.003), and a 4 ± 6% increase after AD in connection with the belt (94 ± 18% vs. 98 ± 21% (*p* = 0.03)).

FEV1 values also increased after AD therapy in combination with both the Simeox device and belt by 6 ± 7% (84 ± 18% vs. 90 ± 16%; *p* = 0.03) (Figure 1b). However, there were no significant differences between FEV1 after AD therapy with a belt or Simeox device (*p* > 0.05). However, following AD in combination with Simeox therapy, the difference in FEV1 values in patients under 10.5 years of age (the median age) was significantly greater (by 8%) vs. older patients (*p* = 0.02) (Table 1). Furthermore, AD therapy with the belt led to significantly greater difference in FEV1 values (by 10%) in patients over 10.5 years of age (*p* = 0.02) (Table 1). 

FEV3 values increased by 7 ± 9% after using AD with the belt and Simeox device, and the difference before and after therapy was found to be statistically significant (96 ± 17% vs. 103 ± 18%; *p* = 0.02). There were no statistically significant differences in FEV3 values following the other therapies (Figure 1c). However, there was difference in FEV3 values (greater than 12%) in children under 10.5 years of age following AD therapy with the belt and Simeox device in comparison to older patients (*p* = 0.02). In addition, the group of patients younger than 10.5 years showed an FEV3 difference of more than 10% (*p* = 0.02) after AD therapy with the belt alone (Table 1).

FEV6 values following AD therapy with a belt and Simeox device were 8 ± 8% (94 ± 18% vs. 102 ± 17%, *p* = 0.009) (Figure 1d). A difference of 5 ± 5% in mean FEV6 values was observed after AD therapy with the belt (91 ± 20% vs. 96 ± 21%; *p* = 0.008). There were no statistically significant differences in FEV6 values after the other therapies. However, a 10% greater difference in FEV6 was observed in children younger than 10.5 years in comparison to older patients (*p* = 0.02) after using AD with the belt and Simeox device (Table 1).

PEF values after AD therapy in connection with the Simeox device significantly increased by 12 ± 21% (84 ± 22% vs. 96 ± 23%; *p* = 0.03), and after AD in connection with the Simeox device and the belt by 11 ± 17% (89 ± 17% vs. 100 ± 22%; *p* = 0.05) (Figure 1e). After AD therapy with the belt, a PEF difference of greater than 9% was observed in patients under 10.5 years of age vs. older patients (*p* = 0.04) (Table 1). Likewise, following AD therapy with the Simeox device, a PEF difference of greater than 24% was observed in patients younger than 10.5 years of age vs. older patients (*p* = 0.008).

An increase of 13 ± 13% in FET values was seen after the use of AD therapy in connection with the belt and Simeox device (84 ± 16% vs. 97 ± 13%; *p* = 0.008). An increase of 23 ± 24% in FET values was also observed after AD (72 ± 27% vs. 95 ± 28%; *p* = 0.007) (Figure 1f). There were no significant differences in FET values after the other therapies.

### 3.2. Analysis of Blood Oxygen Saturation Levels

**Figure 2:**
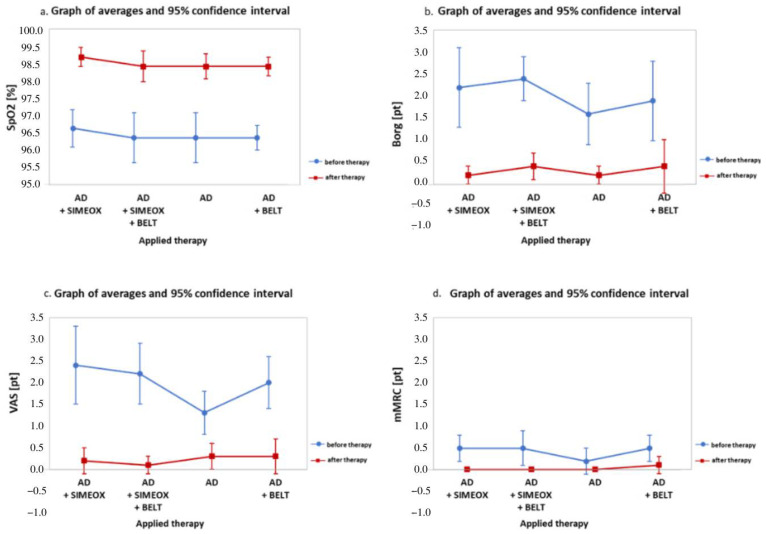
Graphic representation of pre- and post-therapy results for: (**a**) percent blood oxygen saturation (SpO2); (**b**) the Borg Rating of Perceived Exertion Scale (Borg); (**c**) the Visual Analogue Scale (VAS); and (**d**) the modified Medical Research Council (mMRC) dyspnea scale. AD, autogenic drainage.

An increase in blood oxygen saturation was observed after each applied therapy. There was an increase in saturation values over 2% after: AD with the Simeox device (97 ± 1% vs. 99 ± 0%; *p* ≤ 0.0001); AD with a belt and Simeox device (97 ± 1% vs. 99 ± 1%; *p* ≤ 0.0001); AD alone (97 ± 1% vs. 99 ± 1%; *p* ≤ 0.0001); and AD with the belt (97 ± 1% vs. 99 ± 0%; *p* ≤ 0.0001) (Figure 2a). There were no significant differences in saturation in terms of age.

### 3.3. Assessment of Dyspnea and Fatigue

After each therapeutic combination was analyzed, a decrease in scores according to the Borg (Figure 2b), VAS (Figure 2d), and mMRC scales was observed (Figure 2d).

### 3.4. Analysis of Therapy Efficiency Based on the Studied Parameters

**Figure 3:**
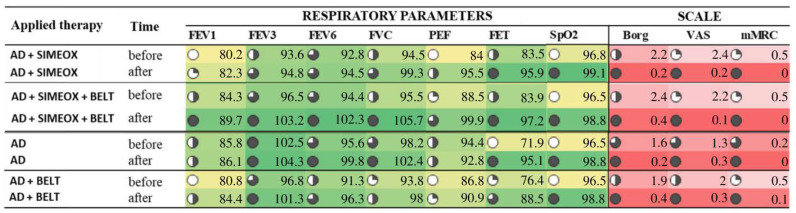
Summary of the mean values of respiratory and circulatory parameters—as well as the Borg, VAS, and mMRC scores—before and after each therapy. Darkened wheels indicate a positive effect; Green represents the highest values of each parameter, yellow represents the lowest values; Red represents the lowest values, indicating a positive effect in the case of the Borg, VAS and mMRC scales. AD—autogenic drainage; FEV1—forced expiratory volume in 1 s; FEV3—forced expiratory volume in 3 s; FEV6—forced expiratory volume in 6 s; PEF—peak expiratory flow; FET—peak expiratory time; SpO2—percent blood oxygen saturation; Borg—the Borg Rating of Perceived Exertion Scale; VAS—the Visual Analogue Scale; mMRC—modified Medical Research Council dyspnea scale.

An analysis of the efficacy of the therapies showed the highest increase in 6 of 7 assessed respiratory parameters following AD therapy in connection with a Simeox device and belt, and 5 out of 7 analyzed respiratory parameters after AD (Figure 3). Each of the therapies resulted in an improvement in the patients’ perception of exertion as assessed by the Borg scale and a decrease in fatigue as assessed by the VAS and mMRC scales.

## 4. Discussion

This study investigated the effectiveness of AD and demonstrated the validity of combining AD therapy with a belt and Simeox device, especially in CF patients under 10 years of age.

During in-hospital treatment of bronchopulmonary disease (hospitalization during an exacerbation of the disease), spirometric parameters are improved by—among other things—the use of antibiotic therapy or intensification of the performed inhalations. In the present study, in contrary to previous ones, a therapy was intentionally applied at the time when patients were not influenced by any of the additional factors affecting the change in respiratory parameters, so that the effect of eachtherapy could be evaluated.

An important parameter analyzed in our study was FVC, as it determines obstructive as well as restrictive lung changes [30,31]. We observed the highest increase in FVC values after AD in combination with the belt and Simeox device, followed by AD in connection with the Simeox device, with the belt, and lastly as a result of AD alone (by 10%, 5%, and 4%, respectively). These observations are in agreement with a previous report showing a significant increase in FVC values of 3 ± 6% 20–30 min following AD therapy in patients with CF over the age of 8 years [16]. 

Current studies on FEV1 in cystic fibrosis show that despite medical advances and an increase in median life expectancy, respiratory failure related (also) to a decrease in the FEV1 parameter remains the leading cause of death among patients with cystic fibrosis [32,33]. Following AD therapy with a belt and Simeox device, and after using the Simeox device alone, an improvement in the FEV1 parameter have been described in patients with CF (by 6% and 9%, respectively) [34]. It is important to monitor the FEV1 parameter because its value < 30% or rapid decline is one of the criteria for putting CF patients on the lung transplant waiting list [35]. However, it should be noted that those patients were supported with the standard treatment of broncho-pulmonary exacerbation, which is in contrast to our study where patients used the standard daily treatment without additionally introduced antibiotics or intensification of inhalations.

One important observation is that the difference in FEV1 values was greater in children younger than 10.5 years after using AD with the Simeox device and AD with the belt (by 8% and 10%, respectively) in comparison to older patients. There are studies on CF patients over the age of 8 years that have shown a 2 ± 5% increase in FEV1 values following AD therapy without the use of assistive techniques [16]. Furthermore, the differences in the results of the FEV1 parameter are not due to a lack of treatment effect or the effectiveness of respiratory therapies in adult CF patients, but to the fact that lung function rises rapidly in childhood and declines slowly in adulthood [36]. This is supported by studies on groups of 6–17-year-old patients that were then repeated in adults divided into two age groups: 18–24 years and ≥25 years [37,38]. Analyses uncovered greater FEV1 decreases in the oldest patients, especially women, and those with additional risk factors (i.e., pancreatic insufficiency, sinusitis, increased sputum production, and *Pseudomonas aeruginosa* bacterial colonization), or increased frequency of broncho-pulmonary disease exacerbations in patients treated with intravenous antibiotic therapy.

An increase in FEV3 and FEV6 levels (by 7% and 7.9%, respectively) was observed after AD therapy with the belt and Simeox device. Moreover, a difference of more than 12% in the FEV3 parameter and 10% in the FEV6 parameter was achieved by patients younger than 10.5 years old vs. older patients. There are no other comparative studies analyzing these parameters in patients with CF. The increase in FEV3 and FEV6 after the therapies, and based on autogenic drainage, may indicate maintenance of the expiratory volume at a high level, even in the third and sixth seconds of the expiratory airflow.

PEF, which can also be an indicator of properly performed spirometry [35], was also analyzed. A significant increase in PEF was observed, by as much as 11%, after AD therapy with the Simeox device and belt, and there was a significant difference (of 24%) between patients under 10.5 years old and those that were older. Thus far, studies among CF patients aged 13–18 years have indicated that the required PEF value is maintained during all phases of AD [39], but does not change after AD therapy [40]. 

We found a 13% increase in the FET value after using AD with a belt and Simeox device. Curiously, the greatest increase in FET—by 23%—was shown following AD alone. The literature does not provide comparisons of this parameter in the context of respiratory therapies applied to CF; from practice, it appears that when performing AD the exhalation is free and the flow takes place without resistance. In AD, with each exhalation, the patient moves to the lowest possible respiratory volume and performs a longer exhalation. Thus, the results showing an increase in expiration time after AD may be related to its use.

In patients with CF, blood oxygen saturation values oscillate around 96% for resting saturation and 94% for post-test saturation [41]. Our results showed an increase in saturation after each of the therapies used. Similarly, in a study by Corten et al. [42], the authors showed that following AD, blood saturation increased from 93% to 95% and was maintained for one hour following therapy. Available studies have also shown that combining therapies, even when they are spaced apart, increases their effectiveness, which also affects the amount of expectorated secretions [34]. Moreover, the VAS, Borg, and mMRC scales confirmed a reduction in fatigue-related discomfort after all of the therapies we investigated. 

One of the main limitations of the present study is the small number of patients. However, it should be noted that there were some exclusion criteria including an unstable condition of the patient or additional medical treatment, apart from routine daily inhalations or pharmacotherapy. Secondly, it was a prospective study in which four types of therapy were compared at the same time in the same patient. The studies of cystic fibrosis patients are usually conducted in similar sized groups [9]. Studies in a larger group of patients are retrospective studies where the respiratory parameters were collected from a database of CF patients [32].

## 5. Conclusions

The application of each respiratory physiotherapy technique based on AD had a positive effect on spirometric parameters and blood oxygen saturation, and reduced fatigue and feelings of dyspnea in patients with CF. The greatest therapeutic effect was observed after AD therapy in connection with the belt and mechanical support for respiratory cleansing. However, to achieve the expected therapeutic effect, the therapy should be conducted by a qualified physiotherapist trained in AD. Given the particular benefits demonstrated in patients under 10.5 years of age, it is important to guarantee real accessibility to this form of physiotherapy, especially in the indicated age group.

## Figures and Tables

**Table 1:** Comparison of the differences in individual therapy parameters by age.

Applied Therapy	Age (Year)	Avg	SD	Min.	Max.	Q25	Me	Q75	*p*-Value
FEV1 difference									
AD + SIMEOX	<10.5	6	4	0	9	6	8	9	**0.02**
AD + SIMEOX	>10.5	−2	5	−8	7	−5	−2	0	
AD + SIMEOX + BELT	<10.5	7	6	1	15	4	5	10	NS
AD + SIMEOX + BELT	>10.5	4	9	−7	19	−2	4	7	
AD	<10.5	1	3	−1	5	−1	−1	4	NS
AD	>10.5	0	4	−7	5	−2	0	2	
AD + BELT	<10.5	9	9	−2	20	3	8	15	**0.02**
AD + BELT	>10.5	−1	3	−6	2	−3	−2	2	
FEV3 difference									
AD + SIMEOX	<10.5	3	5	−4	9	−1	3	7	NS
AD + SIMEOX	>10.5	0	8	−11	10	−5	0	5	
AD + SIMEOX + BELT	<10.5	13	10	4	23	4	11	23	**0.02**
AD + SIMEOX + BELT	>10.5	1	4	−5	7	1	2	3	
AD	<10.5	0	8	−10	8	−9	1	7	NS
AD	>10.5	4	6	−1	15	0	3	5	
AD + BELT	<10.5	10	8	1	24	4	7	12	**0.02**
AD + BELT	>10.5	0	5	−7	4	−5	1	4	
FEV6 difference									
AD + SIMEOX	<10.5	2	7	−8	10	−1	3	6	NS
AD + SIMEOX	>10.5	2	8	−8	11	−5	2	8	
AD + SIMEOX + BELT	<10.5	14	8	5	22	6	15	20	**0.02**
AD + SIMEOX + BELT	>10.5	4	5	−5	8	1	4	7	
AD	<10.5	2	8	−8	14	−5	2	8	NS
AD	>10.5	6	6	0	15	3	5	10	
AD + BELT	<10.5	7	5	1	14	4	7	9	NS
AD + BELT	>10.5	3	6	−6	11	0	4	6	
PEF difference									
AD + SIMEOX	<10.5	25	27	4	71	8	19	21	**0.008**
AD + SIMEOX	>10.5	1	3	−4	4	−1	1	3	
AD + SIMEOX + BELT	<10.5	16	21	−11	40	3	12	34	NS
AD + SIMEOX + BELT	>10.5	8	13	−5	26	−2	5	18	
AD	<10.5	2	17	−27	17	−8	6	15	NS
AD	>10.5	−5	8	−15	9	−8	−7	−1	
AD + BELT	<10.5	9	6	−2	15	6	10	13	**0.04**
AD + BELT	>10.5	0	7	−9	7	−7	1	6	

AD—autogenic drainage; FEV1—forced expiratory volume in 1 s; FEV3—forced expiratory volume in 3 s; FEV6—forced expiratory volume in 6 s; PEF—peak expiratory flow; Avg—Averages; SD—standard deviation; Min.—minimum; Max.—maximum; Me—median; Q25—bottom quartile 25%; Q75—Top quartile 75%; *p*—probability; NS = not significant.

## Data Availability

Not applicable.
